# Characterization of clinical risk factors for enhanced molecular residual disease monitoring in resected early-stage non-small cell lung cancer

**DOI:** 10.1186/s12885-025-15259-6

**Published:** 2025-12-04

**Authors:** Yunfei Shi, Youming Lei, Yinqiang Liu, Wei Zhao, Qingmei Yang, Fujun Zhang, Li Bian, Xiaoyu Luo, Kang Luo, Haoyu Li, Fanghao Liu, Tingguang Wang, Yong Liu, Ya Ma, Jiani C. Yin, Guoli Lv, Jin Duan

**Affiliations:** 1 Department of Thoracic Surgery, The First Affiliated Hospital of Kunming Medical University, 295 Xichang Road, Kunming, 650031 China; 2 Department of Pathology, The First Affiliated Hospital of Kunming Medical University, Kunming, China; 3 Geneseeq Research Institute, Nanjing Geneseeq Technology Inc., Nanjing, Jiangsu China

**Keywords:** Non-small cell lung cancer, Molecular residual disease, Clinical risk factors

## Abstract

**Background:**

Lung cancer is a leading cause of cancer mortality, with non-small cell lung cancer (NSCLC) accounting for the majority of cases. Standard treatment for early-stage NSCLC involves surgical resection, often complemented by adjuvant therapies. This study explores the dynamics of molecular residual disease (MRD) in early-stage NSCLC, emphasizing the prognostic value of baseline clinical characteristics.

**Methods:**

We conducted a prospective study enrolling 78 patients with operable stage I-IIIA NSCLC. Serial blood samples were collected at various postoperative intervals to evaluate MRD via circulating tumor DNA (ctDNA) analysis. Clinical variables, such as tumor stage, driver mutations, carcinoembryonic antigen (CEA) levels, and histopathological characteristics, were assessed for their predictive capabilities for MRD positivity and disease relapse.

**Results:**

Among the 78 patients, 60.3% were diagnosed at stage IA, with a longitudinal MRD positivity rate of 20.5% (16/78). MRD-positive patients demonstrated shorter disease-free survival (DFS) compared to MRD-negative patients (hazard ratio = 171.4, *P* < 0.001). All nine patients with radiological relapse were MRD-positive, with a median lead time from MRD detection to relapse of 242.0 days. MRD + /DFS events were significantly associated with advanced clinical staging, absence of driver mutations, and elevated baseline CEA levels. A clinical risk factor (CRF) scoring system including the three factors effectively stratified patients by relapse risk, identifying those who could benefit from intensified postoperative treatment. The MRD + /DFS event rate was 36.3% for the CRF-high group compared to 0.0% for the CRF-low group (*P* < 0.001). The CRF score also stratified the patients by event-free survival, where CRF-low patients showed more favorable outcome compared to CRF-high patients (hazard ratio = 9.0, *P* < 0.001).

**Conclusions:**

Our findings highlight the interplay between clinical risk factors and MRD dynamics in early-stage NSCLC, offering insights for risk stratification and personalized treatment approaches. The CRF score may help clinicians tailor adjuvant therapies, ultimately improving outcomes for high-risk patients.

**Supplementary Information:**

The online version contains supplementary material available at 10.1186/s12885-025-15259-6.

## Introduction

Lung cancer remains a leading cause of cancer-related deaths worldwide, with non-small cell lung cancer (NSCLC) constituting the most prevalent subtype [1–3]. For patients diagnosed with early-stage NSCLC, the standard treatment protocol is surgical resection, often supplemented by adjuvant chemotherapy. In cases deemed inoperable, the preferred treatment modality is concurrent chemotherapy and radiotherapy. Recent advances in the molecular understanding of NSCLC and drug development have underscored the importance of systemic adjuvant therapies, including chemotherapy, targeted therapies, and immunotherapies, offering potential benefits for patients who might need further intervention to improve clinical outcomes [4, 5]. According to the National Comprehensive Cancer Network (NCCN) guidelines for NSCLC [6], systemic adjuvant therapy is recommended after surgery for patients staged IB and higher. The guidelines also outline several clinical features that elevate the risk of recurrence, such as poor tumor differentiation, vascular invasion, wedge resection, and visceral pleural involvement. However, risk stratification remains challenging, particularly for patients staged IA or IB. As a result, clinicians often rely on their discretion and patient preferences when deciding whether to pursue active surveillance or additional treatments.

Given the challenges in risk stratification for patients with early-stage NSCLC, understanding factors that can influence disease outcomes is critical. Molecular residual disease (MRD) has been extensively investigated in this context, as it correlates with tumor burden and the likelihood of disease relapse [7]. The advent of Liquid biopsy, coupled with the enhanced sensitivity of next-generation sequencing (NGS) for detecting MRD, has enabled the monitoring of NSCLC progression at a molecular level. Detection of circulating tumor DNA (ctDNA) has shown great promise, as ctDNA positivity can indicate potential disease relapse months before it becomes detectable through radiological imaging, with a mean lead time of 5.5 months [8]. This predictive capability positions ctDNA as a potential trial endpoint or a landmark point for guiding therapeutic intervention [9]. Nevertheless, there is still limited systematic investigation into the association between longitudinal MRD positivity and baseline clinical high-risk features [10].

In this study, we conducted a prospective study examining the longitudinal dynamics of MRD in 78 early-stage NSCLC patients who underwent surgical resection. Our objective was to clarify the prognostic significance of baseline clinical features in association with MRD positivity and disease relapse. In addition to the high-risk factors outlined by the NCCN guidelines, we assessed the relevance of common clinical indicators, including the total proportion of solid and micropapillary components (TPSM) of the tumor, the presence of driver mutations, tumor mutational burden (TMB), PD-L1 expression, and carcinoembryonic antigen (CEA) levels. This study aims to enhance our understanding of prognostic evaluations in early-stage NSCLC, potentially contributing to refined risk stratification and treatment strategies.

## Methods

### Patient inclusion and sample collection

Patients with stage I-IIIA operable NSCLC who underwent surgical resection at the First Affiliated Hospital of Kunming Medical University from November 2020 and May 2024 were enrolled in the study. Tumor tissue samples were obtained during surgery. Peripheral blood samples, collected at baseline, during surgery, and at various post-operative time points, including post-operative day (POD) 3–7 and post-operative month (POM) 6, 12, 15, 18, and 21, were analyzed to assess longitudinal changes in MRD levels. White blood cells were isolated from the blood samples to serve as controls for germline variations.

Patient demographic and clinical information, including age, sex, smoking status, clinical stage, metastasis status, tumor histology (e.g., differentiation grade and TPSM), driver mutation positivity, and PD-L1 expression, were documented. Additional clinical factors, such as baseline CEA level, wedge resection and the presence of pleural or lymphovascular invasion, were also recorded. Treatment outcomes were assessed with a median follow-up duration of 459.0 days (95% confidence interval [95%CI], 379.0–489.0 days). Disease-free survival (DFS) was defined as the time from the date of definitive surgery to the first occurrence of radiographic recurrence or death. Event-free survival (EFS) was defined as the time from surgery to either radiographic relapse or positive MRD detection, whichever occurred first.

Written informed consent was obtained from all patients. The observational study adhered to the Declaration of Helsinki, and was approved by the Ethics Committee of the First Affiliated Hospital of Kunming Medical University (No. (2024) ERL No. 259).

### DNA extraction and sequencing

Peripheral blood samples (8–10 ml) were collected in EDTA-coated tubes and centrifuged at 1,800 × g for 10 min within 2 h of blood collection for ctDNA extraction using the QIAamp Circulating Nucleic Acid Kit (Qiagen, Germany). Genomic DNA from fresh tumor tissue or the white blood cells were extracted with the DNeasy Blood and Tissue Kit (Qiagen). Sequencing libraries were prepared by using the KAPA Hyper DNA Library Prep Kit (Roche, Switzerland). The libraries were then enriched for 437 solid tumor-related genes, using probe-based hybridization capture panels (GeneseeqPrime™, Geneseeq Technology Inc.) as previously described [11]. After each step, DNA concentration was quantified by a Qubit Fluorometer (Life Technologies, USA). Sequencing was performed on an Illumina HiSeq4000 platform (Illumina, USA) and carried out in a Clinical Laboratory Improvement Amendments (CLIA)-certified and College of American Pathologists (CAP)-accredited laboratory (Geneseeq Technology Inc.). The raw sequence data have been deposited in the Genome Sequence Archive (Genomics, Proteomics & Bioinformatics 2021) in National Genomics Data Center (Nucleic Acids Res 2022), China National Center for Bioinformation/Beijing Institute of Genomics, Chinese Academy of Sciences (GSA: HRA013877).

### Mutation calling and MRD analysis

FASTQ files were analyzed by Trimmomatic [12] for quality control and low-quality reads were removed. Burrows-Wheeler Aligner [13] was used to map the reads to the human reference genome (hg19). PCR duplicates were then removed with Picard (http://broadinstitute.github.io/picard), and local realignments around indels were performed with the Genome Analysis Toolkit [14] to ensure base quality. Finally, VarScan2 [15] was used to identify single nucleotide variants (SNVs) and indels. Copy-number variants (CNV) and fusion variants were detected using CNVkit [16] and DELLY [17], respectively. Depth ratios of above 2.0 and below 0.6 were considered as CNV gain and CNV loss, respectively, which were assessed to determine driver mutation status. To minimize false positives from clonal hematopoiesis of indeterminate potential (CHIP), mutations recurrently associated with hematopoietic clonal expansions (e.g., *DNMT3A*, *TET2*, *ASXL1*, *JAK2*) were excluded from mutation calling. MRD was defined as positive if any plasma mutations matched those found in the paired tissue sample, or if at least three unique plasma mutations were identified with maximum somatic allele frequencies (MSAF) ≥ 1%. This cut-off was chosen to reduce false positives from clonal hematopoiesis, technical noises, and transient/biologically irrelevant variants [18]. Longitudinal MRD positivity was defined as the detection of positive MRD at any of the six postoperative sampling time point (POD3-7, POM6/12/15/18/21). Driver mutation status was defined based on the detection of activating mutations in *EGFR*, *ALK*, *ROS1*, *BRAF*, *KRAS*, *MET*, *RET*, *ERBB2*, and *NTRK* from sequencing. Patients lacking these alterations were categorized as driver-negative. TMB was evaluated as the number of base substitutions and indels in the coding region of the targeted genes, which included synonymous variations to reduce sampling noise and excluded driver mutations that were over-represented during panel sequencing [11].

The positive-predictive value (PPV) and negative-predictive value (NPV) were calculated as the proportion of positive events, as indicated by MRD positivity, that were true positive, as indicated by radiographic recurrence, and the proportion of negative events that are true negative, respectively [19].

### Statistical analysis

The χ^2^ test was utilized to compared MRD positive rates across groups defined by various clinical features. Student’s t-test or the Kruskal–Wallis test were employed to compare continuous variables between groups, and a Wilcoxon paired signed-rank test was used to evaluate the significance of the lead time from MRD detection to computed tomography (CT)-recognized relapse. Cox proportional hazards regression analysis and Kaplan–Meier (KM) estimation were conducted using the R ‘survival’ and ‘survminer’ packages to analyze the survival outcomes, with log-rank tests used to determine the hazard ratios. Variables with a *P* value ≤ 0.1 in the univariable analysis were included in the multivariable Cox regression model. Clinical risk factor (CRF) scores were calculated based on the presence of adverse pathological and clinical features identified as independent risk factors by multivariable regression analysis; each factor contributed one point, yielding a cumulative score. A two-sided *P* < 0.05 was considered significant for all tests unless indicated otherwise. All statistical analyses and data visualization were performed using the R software (version 4.2.1) and GraphPad Prism software (version 9.0.2).

## Results

### Patient characteristics in association with MRD positivity

**Fig. 1:**
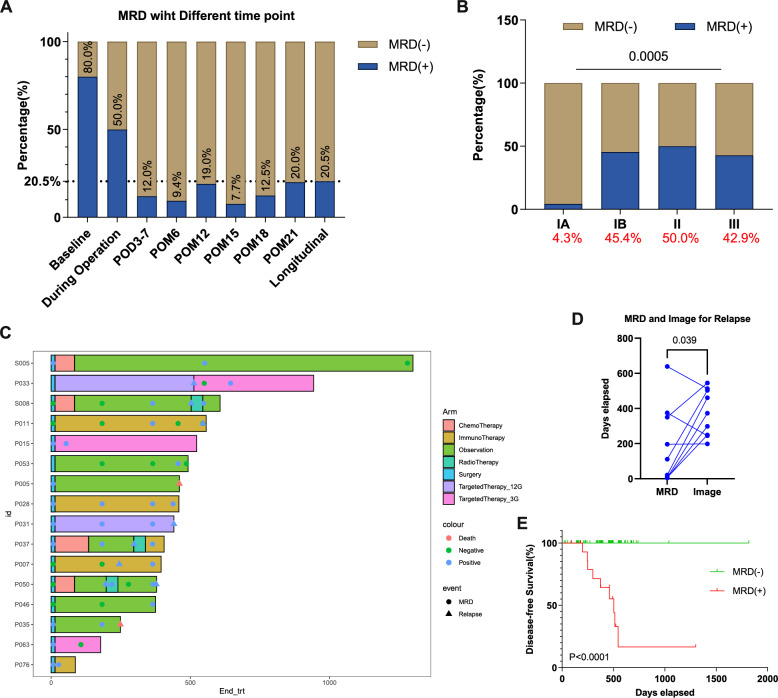
Assessment of longitudinal MRD dynamics in the study cohort. **A** MRD positivity rates at baseline, during operation, and at six post-operative monitoring time points (combined as “longitudinal”) for the NSCLC patients. **B** Significant differences in MRD positivity rates for NSCLC patients of different clinical stages (IA-III) as assessed by the Kruskal–Wallis test (*p* < 0.001). **C** Swimmer plot for the 16 patients exhibiting positive longitudinal MRD, indicating treatment courses and relevant events including MRD positivity, disease relapse, or death, with time 0 representing two weeks post-surgery. **D** Comparison of relapse time measured by MRD and by computed tomography (CT), analyzed using the Wilcoxon paired signed-rank test (*p* = 0.039). **E** Significant difference in disease-free survival for patients with negative and positive longitudinal MRD, analyzed using the log-rank test (*p* < 0.001). MRD, molecular residual disease; POD, post-operative days; POM, post-operative months

A total of 78 patients with resectable NSCLC who underwent surgery were included in our study. Among these patients, 60.3% (47/78) were diagnosed at stage IA, 14.1% (11/78) at stage IB, and 25.6% (20/78) at stage II-IIIA (Table 1). Out of the available baseline plasma samples (*n* = 5), we identified positive MRD (MRD +) in four patients (80.0%) (Fig. 1A). The MRD + rate decreased to 12.0% (9/75) at post-operative day 3–7 (POD3-7) and further declined to 9.4% (5/53) six months after tumor resection (POM6). The MRD + rate was 19.0% (8/42) at POM12, 7.7% (2/26) at POM15, 12.5% (2/16) at POM18, and 20.0% (1/5) at our last follow-up (POM21). The overall longitudinal MRD + rate reached 20.5% (16/78).

**Table 1:** Patient clinical characteristics, overall and grouped by longitudinal MRD positivity

Patient characteristics		MRD-positive (*n* = 16)	MRD-negative (*n* = 62)	*P*-value
Median age (range)		62 (43–76)	56 (26–82)	*p* = 0.419
Sex, n(%)	Male	7 (43.8%)	30 (48.4%)	*p* = 0.786
	Female	9 (56.2%)	32 (51.6%)	
Smoking status, n(%)	Yes	5 (31.2%)	22 (35.5%)	*p* > 0.999
	No	11 (68.8%)	40 (64.5%)	
Histology, n(%)	LUAD	13 (81.2%)	58 (93.6%)	*p* = 0.148
	Non-LUAD	3 (18.8%)	4 (6.4%)	
Lymph node metastasis, n(%)	Yes	4 (25.0%)	10 (16.7%)	*p* = 0.476
	No	12 (75.0%)	50 (83.3%)	
	NA	0	2	
Clinical stage, n(%)	IA (*n* = 47, 60.3%)	2 (12.5%)	45 (72.6%)	*p* < 0.001
	IB (*n* = 11, 14.1%)	5 (31.2%)	6 (9.7%)	
	II-IIIA (*n* = 20, 25.6%)	9 (56.3%)	11 (17.7%)	
Driver mutation, n(%)	Present	11 (68.8%)	56 (90.3%)	*p* = 0.042
	Absent	5 (31.2%)	6 (9.7%)	
TMB, n(%)	≥ 10 mutations/Mb	5 (31.2%)	7 (11.5%)	*p* = 0.113
	< 10 mutations/Mb	11 (68.8%)	54 (88.5%)	
	NA	0	1	
PD-L1 expression, n(%)	≥ 1%	2 (12.5%)	19 (30.6%)	*p* = 0.210
	< 1%	14 (87.5%)	43 (69.4%)	
Clinical Risk Score, n(%)	High Risk	9 (56.2%)	32 (51.6%)	*p* = 0.786
	Low Risk	7 (43.8%)	30 (48.4%)	
TPSM, n(%)	≥ 5%	5 (50.0%)	29 (56.9%)	*p* = 0.738
	< 5%	5 (50.0%)	22 (43.1%)	
	NA	6	11	

Percentages are based on available data. Subgroup totals may not add up to the overall cohort size due to missing information

*LUAD* lung adenocarcinoma, *MRD* molecular residual disease, *TMB* tumor mutational burden, *TPSM* total proportion of solid and micropapillary components, *NA* not available

Comparisons of clinical features between patients with positive longitudinal MRD + status (*n* = 16) to those with negative MRD status (MRD-, *n* = 62) (Table 1) revealed significant differences in clinical stage and the presence of driver mutations. The MRD + group was associated with more advanced stages compared to the MRD- group (*P* < 0.001). The longitudinal MRD + rate was only 4.3% for stage IA patients, contrasting with rates of 45.4%, 50.0% and 42.9% for patients with stage IB, II and III NSCLC, respectively (*P* < 0.001) (Fig. 1B). Of the two stage IA patients with positive MRD, both underwent wedge resection surgeries. In one case, the patient received neoadjuvant therapy, and yet developed brain metastasis 17.0 months following surgery. The other case presented with multiple nodules and exhibited positive MRD at POD3-7. Subsequently, the patient underwent a resection of the contralateral multifocal lesions, ultimately resulting in a negative MRD status. In addition, the MRD + group exhibited a higher proportion of patients without driver mutations compared to the MRD- group (*P* = 0.042). No difference was observed between the MRD + and MRD- patients comparing factors such as TMB, TPSM, and PD-L1 expression levels (Table 1).

### Longitudinal MRD monitoring and disease relapse

The treatment course for the 16 patients with positive longitudinal MRD is documented in Fig. 1C. Among these patients, nine experienced disease relapses, while the remaining seven showed MRD positivity but had not developed radiographic or clinical relapse at the last follow-up.

The median lead time from MRD positivity to imaging-confirmed recurrence was 242.0 days (*P* = 0.039) (Fig. 1D). Two of the relapsed patients showed MRD positivity after relapse. Of these, one had developed brain metastasis, while the other had undergone tumor resection following neoadjuvant therapy, and yet progressive micrometastases were identified in both lungs post-surgery.

Among the seven patients who did not experience relapse, five received adjuvant therapy. The therapy was given either according to guideline-based indications (e.g., stage IB–IIIA NSCLC with driver mutations) or due to the combination of MRD positivity and additional high-risk pathological factors, including vascular invasion, multiple nodules, or airway/pleural spread. MRD dynamics during therapy varied: three patients remained MRD-positive (e.g., P028, treated with immunotherapy, Fig. 1C), whereas two converted to MRD-negative after therapy (e.g., P063, treated with adjuvant osimertinib). The other two patients who did not receive adjuvant therapy were initially MRD- post-surgery but later converted to MRD + at the last follow-up. These findings suggest that adjuvant therapy may influence MRD status, but clearance is not achieved in all cases.

In summary, the sensitivity of longitudinal MRD positivity for predicting disease relapse were 100.0% (95% CI: 66.4% to 100.0%) at a specificity of 89.9% (95% CI: 80.2% to 95.8%). The positive and negative predictive values were 56.3% (95% CI: 38.9% to 72.2%) and 100.0% (95%CI: 94.2% to 100.0%), respectively.

In line with the high sensitivity of longitudinal MRD positivity to predict disease relapse, we found that MRD status effectively predicts patient DFS. MRD + patients experienced significantly shorter DFS compared to MRD- patients (mDFS = 503.0 days vs not reached, hazard ratio [HR] = 171.4, 95% CI = 32.8–894.4, *P* < 0.0001) (Fig. 1E). Consequently, we propose utilizing MRD positivity as an alternative outcome measure alongside DFS. Given that all patients who relapsed also tested positive for MRD, we further refined our outcome measures by combining MRD status and DFS. For subsequent analyses, we considered either MRD positivity or disease relapse, whichever occurred first, as the end-point, with a primary focus on exploring associations with clinical high-risk factors.

### Clinical risk factors regarding MRD or DFS

The overall event occurrence rate for either MRD positivity or disease relapse (MRD +/DFS) was 20.5% (16/78). Among those who experienced an MRD +/DFS event, we observed 93.8% (15/16) of such events within 18 months after surgery, with only one patient (6.3%) experiencing MRD +/DFS event beyond 18 months (Figure S1**−**2). During the POM0-6 period, five MRD + events were recorded, with relapse confirmed in three patients. The remaining two non-relapsed patients received targeted adjuvant therapy and immunotherapy, respectively. Notably, the MRD status for the patient receiving targeted adjuvant therapy turned negative.

**Fig. 2:**
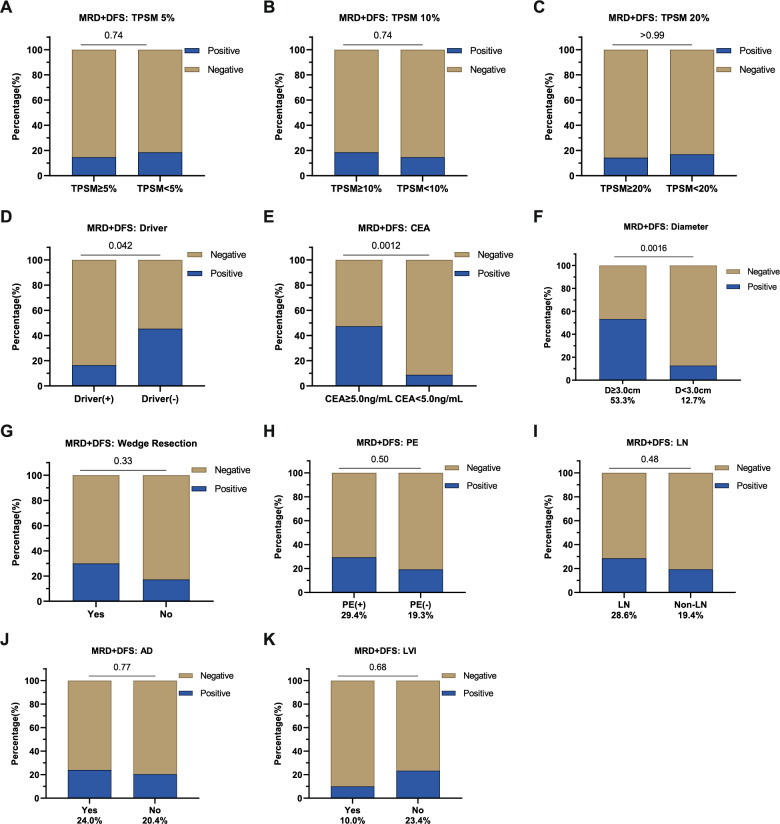
Comparisons of MRD +/DFS event rates among patients with or without various clinical high-risk features. **A**-**C** No significant differences in the event rates comparing patients with higher or lower total proportion of solid and micropapillary components (TPSM) values, using **A** 5%, **B** 10% and **C** 20% as thresholds. Χ^2^ test, *p* > 0.05. **D**-**F** Significant differences in event rates comparing patients different **D** driver mutation statuses (Driver), **E** carcinoembryonic antigen (CEA) levels, and **F** tumor diameters, *p* < 0.05. **G**-**K** No significant differences in the event rates comparing patients with or without **G** wedge resection, **H** pleural invasion (PE), **I** lymph node metastasis (LN), **J** airway dispersal (AD) and **K** lymphovascular invasion (LVI), *p* > 0.05

We further assessed the association between various clinical high-risk factors and MRD +/DFS event rates (Figure S3). In addition to aforementioned clinical stages (*P* < 0.001) and the presence of driver mutations (*P* = 0.042), other potential prognostic risk factors included CEA level and tumor diameter. The event rate was notably higher for patients with a CEA level ≥ 5.0 ng/mL (*P* = 0.001) or a tumor diameter ≥ 3.0 cm (*P* = 0.002) (Fig. 2). On the other hand, factors like TPSM, wedge resection, and pleural or lymphovascular invasion did not show similar prognostic potential. Due to the limited number of cases with poorly differentiated tumors, this factor was excluded from further analyses. In addition, as clinical staging already accounts for tumor diameter, subsequent analysis focused on staging, CEA levels, and driver mutation status.

### Multivariable analysis and development of the CRF score

**Fig. 3:**
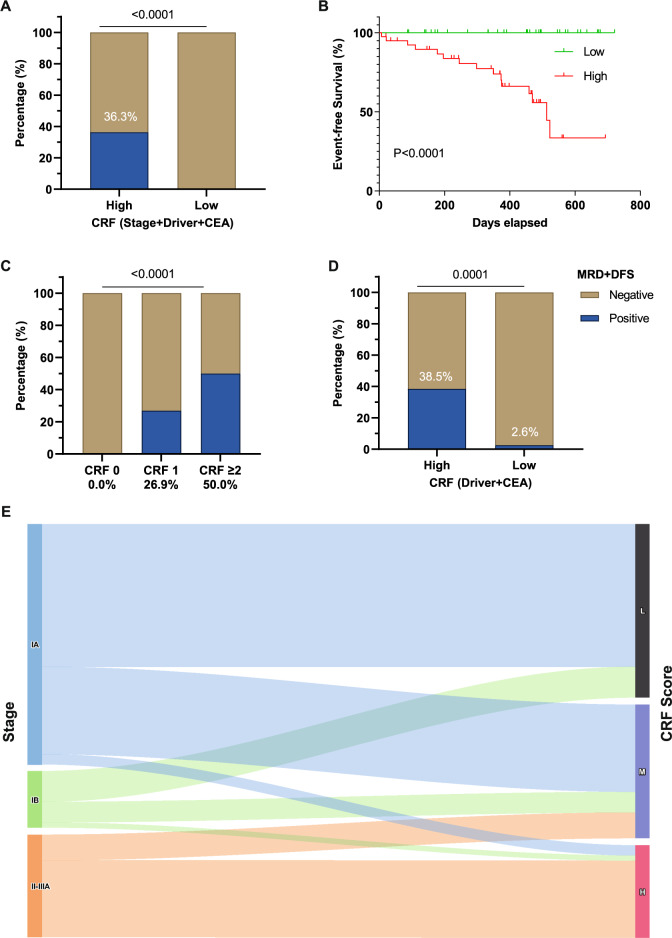
Association of the clinical risk factor (CRF) score with patient outcomes. **A** Significant difference in the MRD +/DFS event rates for patients with high or low CRF scores, as assessed by the Χ^2^ test (*p* < 0.001). A low CRF score is assigned to patients with none of the three high-risk features, while others are assigned to the high CRF group. CEA, carcinoembryonic antigen. **B** Significant difference in event-free survival for patients with high and low CRF scores, analyzed using the log-rank test (*p* < 0.001). **C** Significant difference in the MRD +/DFS event rates for patients with 0, 1, or ≥ 2 CRF scores, with each high-risk feature (stage II-III, absence of driver mutations, carcinoembryonic antigen level > 5 ng/mL) counting as 1 point. Χ^2^ test, *p* < 0.001. **D** Significant difference in the MRD +/DFS event rates for patients with either driver mutations or high CEA levels (High) versus those with neither of these high-risk features (Low), assessed using the Χ^2^ test (*p* < 0.001). **E** Sankey diagram showing the proportions of patients of different clinical stages (IA, IB, II-IIIA) assigned to three groups based on high (H, CRF ≥ 2), medium (M, CRF = 1), or low (L, CRF = 0) risk scores

A combined risk assessment was conducted to evaluate the prognostic potential of CRFs, including advanced staging (stage II-IIIA), high CEA levels (≥ 5.0 ng/mL), and absence of driver mutations. Patients were divided into two groups: CRF-high, consisting of individuals with at least one of the CRFs, and CRF-low, which included those without any of these CRFs. Patients in the CRF-low group exhibited an MRD +/DFS rate of 0.0% as of last follow-up, compared to a rate of 36.3% for the CRF-high group (*P* < 0.001) (Fig. 3A). Similarly, CRF status effectively stratified patients by event-free survival based on MRD +/DFS, which was markedly prolonged in the CRF-low group (mDFS = 513.0 vs not reached, HR = 9.0, 95% CI = 3.2–25.2, *P* < 0.001) (Fig. 3B).

To further refine the risk stratification, we classified patients by their CRF scoring into three groups, “CRF 0”, “CRF 1”, and “CRF ≥ 2”, with “CRF 0” indicating the lowest risk. Of the entire cohort, 43.6% of the patients (34/78) had a CRF score of 0, while 33.3% (26/78) and 23.1% (18/78) had CRF scores of 1 and 2, respectively. We observed an increasing trend of MRD +/DFS event rates associated with increasing CRF scores, with no events in the “CRF 0” group, escalating to 26.9% for the “CRF 1” group and 50.0% for the “CRF 2” group (χ^2^ test, *P* < 0.001) (Fig. 3C).

We also examined the relationship between CRF scoring and clinical staging (Fig. 3E and Table S1). In our cohort, 60.0% of the patients presented with stage IA, 15.0% with stage IB, and 25% with stage II-IIIA NSCLC. In the stage IA group, 59.6% had a CRF score of 0, while 36.2% and 4.2% had scores of 1 or 2, respectively. Stage IB patients displayed a similar distribution, although with a slightly higher rate of 9.2% with a CRF score ≥ 2. In more advanced stages (II-IIIA), all patients had a CRF score of at least 1, and notably, 75.0% of them had a CRF score ≥ 2.

Finally, we conducted Cox regression analyses with the CRF score included as a clinical factor (Table 2). Univariable analysis identified age (*P* = 0.053), clinical stage (*P* < 0.001), CEA level (*P* < 0.001) and CRF score (*P* < 0.001) as significant prognostic factors. In addition, driver mutation status, with a univariable *P* value of 0.100, was also included in the multivariable analysis. Multivariable analysis revealed that driver mutation status (*P* = 0.045) and CEA levels (*P* = 0.001) were independent factors influencing patient prognosis, prompting further examination of patients with these two risk factors (Fig. 3D). Only 2.6% of patients exhibiting both a driver mutation and low CEA levels (< 5 ng/mL) experienced MRD positivity or disease relapse. Conversely, the MRD +/DFS event rate increased to 38.5% for patient exhibiting the absence of driver mutations or high CEA levels (*P* < 0.001). The predictive value for the combination of driver mutation status and CEA levels showed a sensitivity of 93.8% (69.8% to 99.8%) and a specificity of 61.3% (48.1% to 73.4%). PPV and NPV were 38.5% (30.8% to 46.7%) and 97.4% (84.9% to 99.6%), respectively.

**Table 2:** Univariable and multivariable Cox analysis comparing the event-free survival for patients of different clinical features

Variable	Univariable analysis		Multivariable analysis	
	**HR (95% CI)**	***P*-value**	**HR (95% CI)**	***P*-value**
Age, years				
> 60 versus ≤ 60	2.78 (0.99 ~ 7.92)	0.053	1.08 (0.31 ~ 3.78)	0.902
Gender				
Male versus Female	0.93 (0.33 ~ 2.62)	0.891		
Pathological stage				
II-III versus I	6.32 (2.23 ~ 17.91)	< 0.001	3.31 (0.86 ~ 12.69)	0.081
Driver gene				
Negative versus Positive	2.42 (0.82 ~ 7.27)	0.100	4.51 (1.03 ~ 19.72)	0.045
CEA				
≥ 5.0 ng/mL versus < 5.0 ng/mL	11.36 (3.05 ~ 42.38)	< 0.001	12.39 (2.67 ~ 57.41)	0.001
CRF Score				
≥ 1 versus 0	8.04 (2.80 ~ 23.08)	< 0.001	NA	NA

Variables with a *P* value ≤ 0.1 in the univariable analysis were included in the multivariable Cox regression model

*CEA* carcinoembryonic antigen, *CI* confidence interval, *CRF* clinical risk factors, *HR* hazard ratio, *NA* not applicable

## Discussion

Our study provides valuable insights into the longitudinal dynamics of MRD in early-stage NSCLC patients following surgical resection, placing particular emphasis on its association with baseline clinical risk factors. To the best of our knowledge, our findings are the first to reveal key clinical risk factors, including clinical staging, driver mutation status, and CEA levels, that effectively predicts MRD positivity and DFS events.

Longitudinal MRD changes following early-stage NSCLC resection, especially in the context of ctDNA dynamics, have been the subject of extensive research, especially concerning their association with postoperative DFS. In our study, we observed a longitudinal MRD + rate of 20.5% (16/78), which is nearly double the MRD + rate at POD3-7 (12.0%, 9/75). Our findings align with a growing body of literature exploring MRD changes in the context of ctDNA dynamics following NSCLC resection. As reviewed by Verzè et al., MRD + rates in prior studies have shown significant variability, with pre-operative rates ranging from 18.3% to 100.0%, post-operative rates ranging from 6.4% to 46.2%, and longitudinal relapse rates ranging from 14.0% to 58.0% [8]. The heterogeneity observed in these studies is likely influenced by factors such as disease stage distributions, postoperative sampling timepoints, and methodological differences. Nonetheless, the trend of decreasing MRD positivity immediately following surgery, followed by a gradual increase correlating with disease relapse, is consistent with our findings. In prior studies, post-operative samples are typically collected within 30 days following surgery [8]. In addition, ctDNA status at POD3 or POD30 was found to be more significantly correlated with patient DFS than that at POD1 [20]. Gale et al. sampled patient plasma at POD1-3, revealing a post-operative ctDNA + rate of 25.0%, similar to their longitudinal ctDNA + rate of 26.0% [2]. In our study, the lower MRD + rate at POD3-7 compared to the overall MRD + rate reinforces the notion that post-surgical monitoring should commence within days post-surgery to better reflect surgical effects. The fluctuation in MRD + rates at POM6-15 may be attributed to the influences of adjuvant treatments received by certain patients. Importantly, our study demonstrates a high sensitivity and NPV of 100.0% for MRD in predicting disease relapse. All 16 patients who experienced radiographic recurrence had previously shown positive longitudinal MRD. Notably, the median lead time from MRD detection to imaging-confirmed recurrence was 8.1 months, surpassing previously reported lead times of 3.4 to 7.1 months [2, 8, 21]. Conversely, our specificity (89.9%) and PPV (56.3%) align with existing literature [21], and are likely constrained by our relatively short follow-up period of 459.0 days. Specificity and PPV should therefore by interpreted with caution, as this follow-up duration may not have been sufficient to capture all of progression events.

In addition to longitudinal MRD analysis, our study elucidates several clinical features, including clinical staging, driver mutation status, and CEA level, as significant factors that impact MRD +/DFS event rates. While clinical staging has been associated with shorter post-operative DFS, its association with MRD positivity has been rarely explored. In the study by Qiu et al. [18], patients in the T4 stage exhibited significantly shorter DFS compared to those in T1-3 stages, whereas the overall pTMN stage or N stage alone did not differentiate DFS outcomes. In our study, we identified both pTMN stage and tumor diameter (the primary determinant of T staging) as significant predictors of MRD +/DFS event rates. Patients with stage IA disease exhibited a low event rate of 4.3% (2/78), while those with stage IB disease had an event rate of 45.4%, comparable to stage II-III patients (42.9%−50.0%). This suggests that stage IB patients could potentially derive considerable benefit from adjuvant treatments. Notably, both MRD + stage IA patients underwent wedge resection, a less radical surgical approach associated with a higher risk of local recurrence [22, 23]. Though wedge resection did not significantly correlate with MRD positivity across the entire cohort, it is plausible that its role in these specific cases warrants further investigation. It is also noteworthy that high-risk clinical features indicated by the NCCN guidelines, such as lymphovascular or pleural invasion, did not differentiate MRD +/DFS rates within our cohort. Similarly, while TPSM has been associated with post-operative MRD positivity in a preliminary study [10], our findings did not support this association. We propose that these clinical high-risk factors may serve as intermediaries, potentially assessable uniformly through clinical staging.

Absence of driver mutations emerged as an important adverse risk factor in our cohort, correlating with high MRD +/DFS event rates. Clinically, the lack of targetable mutations limits access to effective targeted therapies [24]. In this study, patients without driver mutations received adjuvant immunotherapy or chemotherapy, whereas those with driver mutations had access to targeted therapies, such as osimertinib. Differences in the efficacy of these treatment modalities may partly account for the higher relapse rates among driver-negative patients despite similar stage [25–28]. Nevertheless, it is also plausible that driver mutation status may independently influence tumor behavior. Specifically, common driver mutations, such as *EGFR* and *KRAS* mutations, have been found to be predominantly clonal, while failure to identify a driver clone may indicate a tumor composed of multiple subclones of different mutational origins without a dominant driver clone [3]. This subclonal architecture can increase intratumoral heterogeneity, leading to poor prognosis and elevated relapse risk [3, 29, 30]. It is also plausible that underlying tumor biology not captured by canonical hotspot drivers, such as epigenetic dysregulation or pathway-level activation, can increase genomic instability and ctDNA shedding, thereby heightening MRD detectability and relapse risk. These hypotheses warrant prospective validation and mechanistic study.

CEA levels, a commonly used biomarker in tumor detection, also serves as an independent predictor of MRD +/DFS event rates in our study. Higher CEA levels have been associated with shorter DFS [31]. However, previous studies have noted that CEA alone has suboptimal sensitivity for predicting disease relapse [32, 33]. To enhance sensitivity, a combination of CEA and other molecular markers, such as ctDNA, has been recommended [32]. Previous studies have also utilized radiological progression, ctDNA positivity, or an elevation in CEA levels—whichever manifested first—as indicators for treatment intervention [9]. In our study, CEA levels significantly differentiated MRD + DFS event rates. However, its sensitivity for indicating disease relapse was only 50.0%, aligning closely with a previously reported sensitivity of 49.0% [32]. Our findings highlight the improved predictive capability when combining CEA levels with other risk factors such as driver mutation status. Specifically, utilizing a CRF scoring system, factoring in stage, driver mutation status, and CEA levels, yielded a more comprehensive risk stratification. Our data demonstrated that even patients traditionally considered low-risk (e.g., stage IA or IB) might be at a medium or high risk for relapse when assessed through this scoring framework.

Despite these important findings, our study is not without limitations. First, not all patients provided plasma samples at each of the six monitoring timepoints, which may introduce bias into our longitudinal MRD positivity rates. Second, the preliminary nature of our findings regarding disease relapse rates is constrained by a follow-up period of 459.0 days, and several MRD-positive patients had not yet relapsed by the last follow-up. Therefore, the positive predictive value observed in this cohort should be interpreted with caution, as the true PPV may increase with longer surveillance. We expect that updated data will further substantiate its predictive potential. Thirdly, we adopted a relatively stringent MRD-positivity threshold (≥ 3 mutations with MSAF ≥ 1%, or detection of matched tissue mutations). While this approach minimizes false-positive detection, it may reduce sensitivity in patients with low tumor burden, particularly those with early-stage disease, in whom ctDNA is expected to be exceedingly scarce. To mitigate this, our parallel “matched tissue variant” criterion partially offsets this limitation by permitting MRD calls at lower plasma allele fractions when pre-identified tumor variants are detected, leveraging orthogonal confirmation to maintain specificity. Furthermore, although baseline CEA was an independent predictor of MRD positivity and DFS events, postoperative CEA levels were not consistently collected in our cohort. Thus, we were unable to assess dynamic perioperative changes in CEA levels. Prospective studies incorporating serial CEA measurements are needed to validate their potential prognostic value in conjunction with MRD. Last, given the modest sample size, limited number of candidate covariables, and the data-driven weighing of factors, the prognostic value of the CRF score may be subject to overfitting, and external validation in a large independent cohort is warranted.

In conclusion, our study highlights critical baseline clinical risk factors in NSCLC that can aid in predicting longitudinal MRD dynamics and relapse events. Our findings serve to better identify high-risk patients post-surgery, potentially guiding more personalized treatment strategies. Importantly, by focusing on characteristics associated with favorable prognoses, clinicians can discern which patients require more intensive post-operative attention and which patients may have a favorable prognosis, thus potentially reducing the need for close monitoring. Particularly, early-stage NSCLC patients with driver mutations and lower CEA levels may be candidates for conservative management, while those with adverse indicators may benefit from intensified treatment efforts. We hope that these insights will contribute to enhanced risk stratification and ultimately lead to improved outcomes for early-stage NSCLC patients, assisting clinicians in optimizing post-operative management.

## Supplementary Information

Supplementary Material 1: Figure S1. Change in the number of positive MRD or disease relapse (MRD +/DFS) events at four post-surgical time periods and the cumulative total relapse rate. POM, post-operative months. Figure S2. Positive MRD or disease relapse (MRD +/DFS) event rates at four post-surgical time periods and combined as a longitudinal rate. POM, post-operative months. Figure S3. Significant difference in the positive MRD or disease relapse (MRD +/DFS) event rates for patients exhibiting various clinical high-risk features, *p* = 0.005. Max. diameter > 3, tumor maximum diameter ≥ 3.0 cm; Baseline CEA > 5, baseline carcinoembryonic antigen level ≥ 5.0 ng/mL. Table S1. Exact percentages of patients of different clinical stages (IA, IB, II-IIIA) assigned to three groups based on their clinical risk factor (CRF) scores.

## Data Availability

All data analysed during this study are included in this published article and its supplementary information files. The raw sequence data reported in this paper have been deposited in the Genome Sequence Archive (Genomics, Proteomics & Bioinformatics 2021) in National Genomics Data Center (Nucleic Acids Res 2022), China National Center for Bioinformation/Beijing Institute of Genomics, Chinese Academy of Sciences (GSA: HRA013877) that are available for review at https://ngdc.cncb.ac.cn/gsa-human/s/Qa8q05JR.

## Abbreviations

CEA: Carcinoembryonic antigen
CHIP: Clonal hematopoiesis of indeterminate potential
CI: Confidence interval
CRF: Clinical risk factor
CT: Computed tomography
ctDNA: Circulating tumor DNA
DFS: Disease-free survival
EFS: Event-free survival
HR: Hazard ratio
KM: Kaplan–Meier
MRD: Molecular residual disease
MSAF: Maximum somatic allele frequencies
NCCN: National Comprehensive Cancer Network
NGS: Next-generation sequencing
NPV: Negative-predictive value
NSCLC: Non-small cell lung cancer
POD: Post-operative day
POM: Post-operative month
PPV: Positive-predictive value
TMB: Tumor mutational burden
TPSM: Total proportion of solid and micropapillary components
