# Supplementary material for: Characterization of clinical risk factors for enhanced molecular residual disease monitoring in resected early-stage non-small cell lung cancer
Source: BMC Cancer. 2025 Dec 4;26:93. doi: 10.1186/s12885-025-15259-6 (PMC12821982; doi:10.1186/s12885-025-15259-6)
Supplement: Supplementary file 1 — Supplementary Material 1: Figure S1. Change in the number of positive MRD or disease relapse (MRD +/DFS) events at four post-surgical time periods and the cumulative total relapse rate. POM, post-operative months. Figure S2. Positive MRD or disease relapse (MRD +/DFS) event rates at four post-surgical time periods and combined as a longitudinal rate. POM, post-operative months. Figure S3. Significant difference in the positive MRD or disease relapse (MRD +/DFS) event rates for patients exhibiting various clinical high-risk features, p = 0.005. Max. diameter > 3, tumor maximum diameter ≥ 3.0 cm; Baseline CEA > 5, baseline carcinoembryonic antigen level ≥ 5.0 ng/mL. Table S1. Exact percentages of patients of different clinical stages (IA, IB, II-IIIA) assigned to three groups based on their clinical risk factor (CRF) scores. [file 12885_2025_15259_MOESM1_ESM.pdf]

Figure S1. Change in the number of positive MRD or disease relapse (MRD+/DFS) events at four post-surgical time periods and the cumulative total relapse rate. POM, post-operative months.

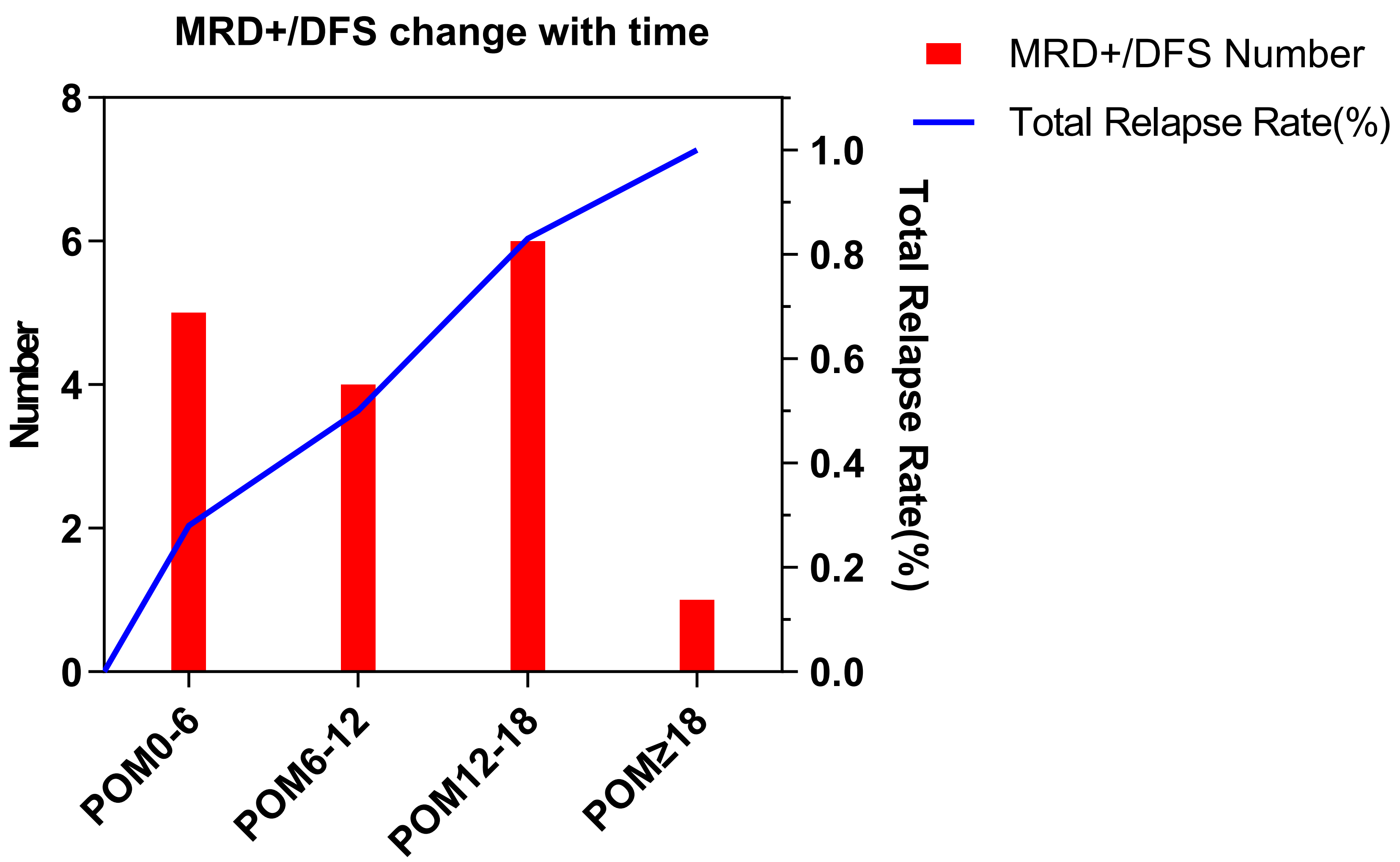

Figure S2. Positive MRD or disease relapse (MRD+/DFS) event rates at four post-surgical time periods and combined as a longitudinal rate. POM, post-operative months.

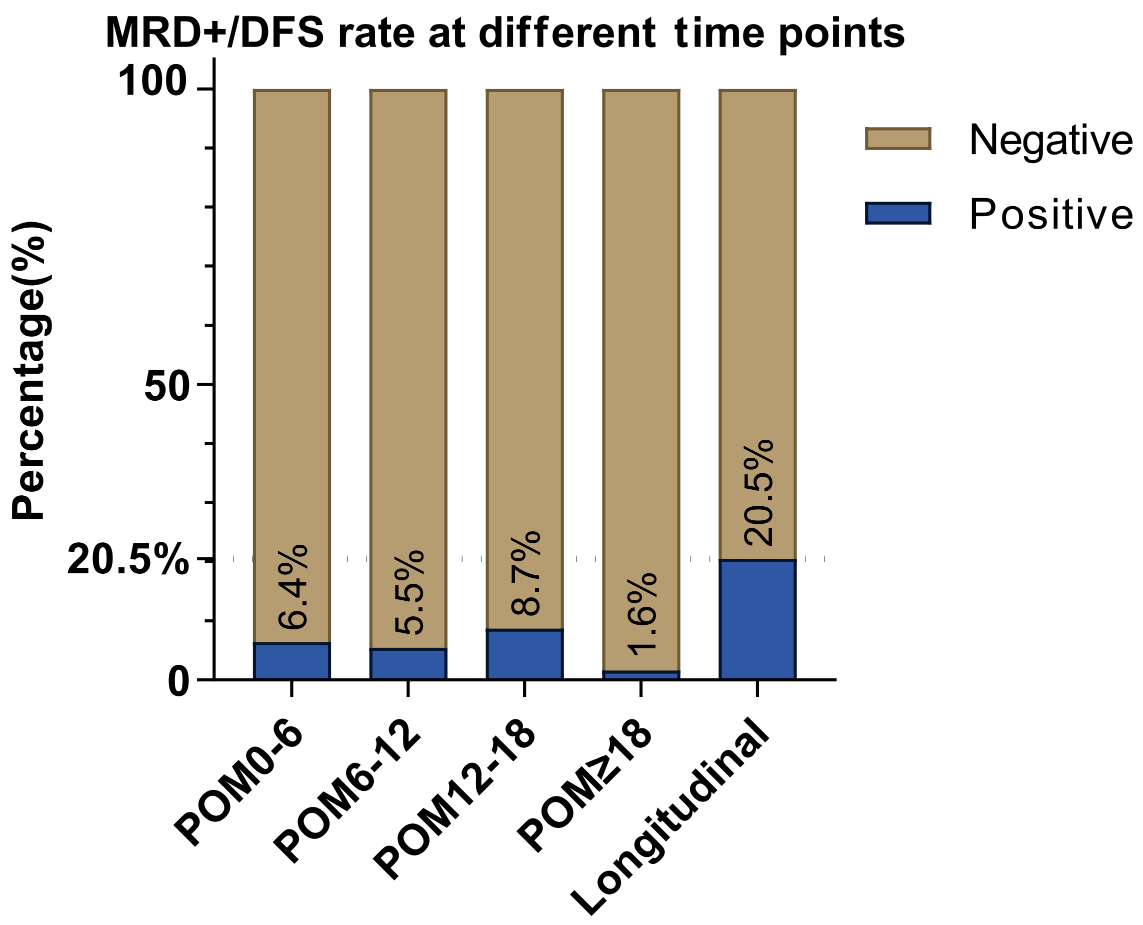

Figure S3. Significant difference in the positive MRD or disease relapse (MRD+/DFS) event rates for patients exhibiting various clinical high-risk features, p=0.005. Max. diameter>3, tumor maximum diameter ≥ 3.0 cm; Baseline CEA>5, baseline carcinoembryonic antigen level ≥ 5.0 ng/mL .

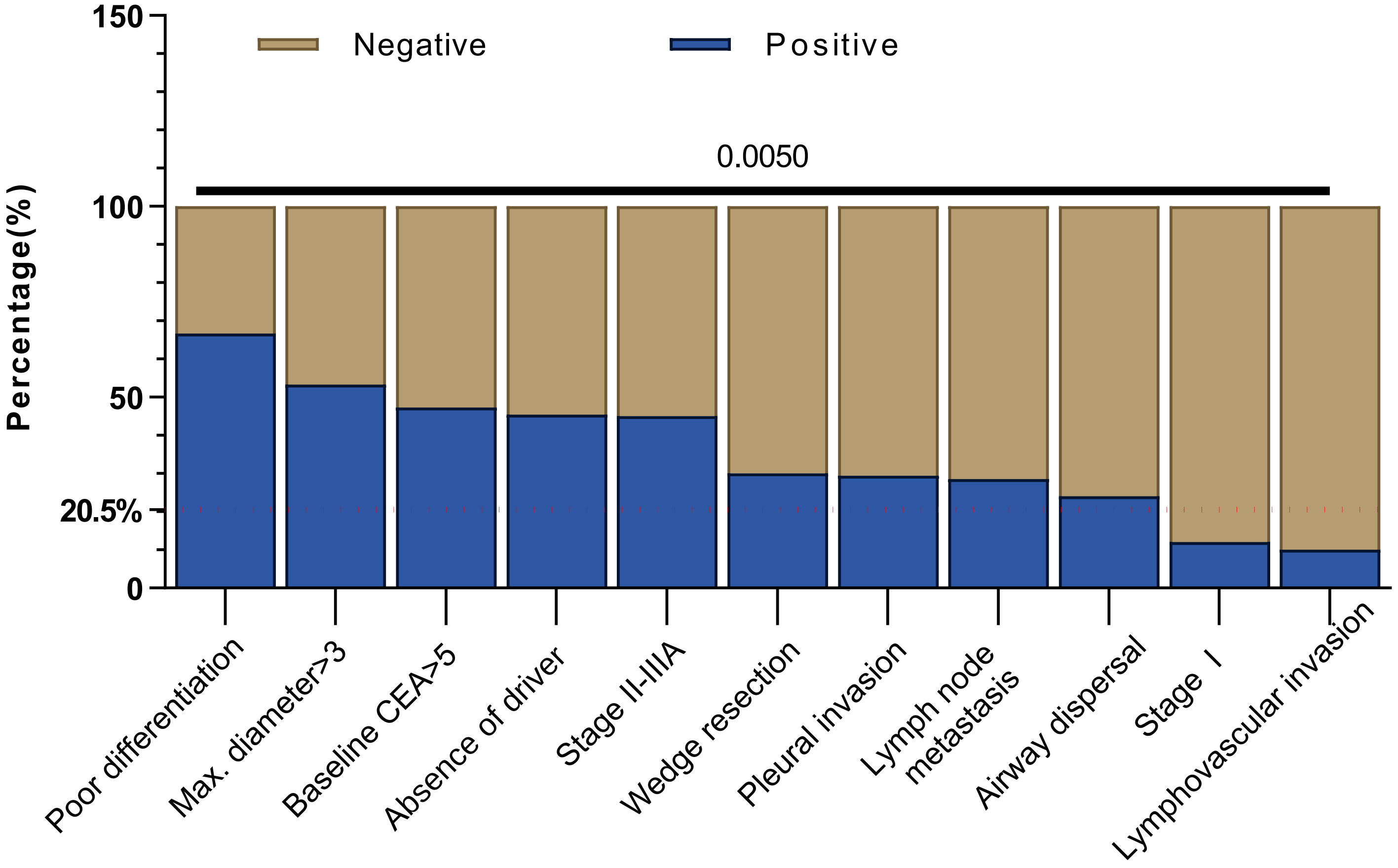

Table S1. Exact percentages of patients of different clinical stages (IA, IB, II-IIIA) assigned to three groups based on their clinical risk factor (CRF) scores.

Stage	CRF0	CRF1	CRF≥2
**IA**	59.6%	36.2%	4.2%
**IB**	54.5%	36.4%	9.1%
**II-IIIA**	0.0%	25.0%	75.0%
